# Does the interface between individual 3D acetabular implants and host bone influence the functional outcomes in patients with severe bone loss after revision surgery?

**DOI:** 10.1051/sicotj/2024040

**Published:** 2024-10-24

**Authors:** Valery Yu. Murylev, Grigory A. Kukovenko, Olga Efimenko, Aleksei V. Muzychenkov, Pavel M. Elizarov, Alexander I. Rudnev, Semyon S. Alekseev, Dmitrii O. Golubkin

**Affiliations:** 1 First Moscow State Medical University named after I.M. Sechenov (Sechenov University), Ministry of Health of Russia St. Trubetskaya, 8/2 119991 Moscow Russia; 2 GBUZ City Clinical Hospital. S.P. Botkin of the Moscow Department of Health 2nd Botkinsky pr-d, 5 125284 Moscow Russia; 3 Limited liability company «TIOS» (LLS «TIOS») Novatorov Street, 6 119421 Moscow

**Keywords:** Custom-designed components, Hip, Arthroplasty, Bone loss, Revision hip arthroplasty, Custom-designed 3D acetabular implants, Positioning, Computed tomography

## Abstract

*Introduction*: There is a wide range of commercially produced revision implants for adequate reconstruction of acetabular large bone defects today, however, it is not always possible to achieve long-term survival of these implants. There is an increasing number of scientific publications concerning the use of custom-designed 3D components, which make it possible not only to achieve stable fixation and connect the pelvic bones but also to restore hip joint biomechanics. *Objectives*: To evaluate the positioning of 3D acetabular implants after revision hip arthroplasty and its impact on clinical and functional outcomes. *Methods*: we analyzed results in 48 patients with bone defect types IIIA and IIIB Paprosky types, after revision hip arthroplasty. A prospective study was conducted from 2017 to 2023. Revision arthroplasty due to aseptic loosening of the components was performed in 30 cases and as a second stage of periprosthetic infection treatment in 18 cases. *Results*: We did not achieve a statistically significant difference when using additional flanges and clinical and functional results. In 2 cases we faced aseptic loosening in patients using flanges. In no case were we able to install an implant with 100% adherence to porous structure compared to preoperatively planned adherence. According to the WOMAC and VAS scales, increasing the contact area of the components showed a slight statistical difference in the improvement of clinical and functional results and the reduction of pain. *Conclusions*: When acetabular 3D components adhered to the bone by more than 68%, we did not register a single complication in the postoperative period, and acetabular 3D components adhered to the bone by less than 68%, a total of 8 (16.6%) complications were registered.

## Introduction

Due to the increased use of primary arthroplasty worldwide, the need for revision arthroplasty is accordingly growing [1]. A rather important point is the preoperative planning of the extent of revision arthroplasty; correct classification of bone defects in the acetabular area is required for this purpose [2]. The classification of bone defects of the acetabulum proposed in 1994 by W.G. Paprosky is most commonly used among orthopedic traumatologists. Thus, type IIIA and type IIIB defect reconstruction with possible pelvic bone dissociation is a difficult task for the surgeon; considering that, the larger the defect, the higher the risk of failure after revision [3–5].

Today there is a wide range of commercially produced revision implants for adequate reconstruction of acetabular large bone defects, however, it is not always possible to achieve long-term survival of these implants [2, 6]. In the past, anti-protrusion ring implantation was the preferred method for type IIIB acetabular bone defects, however, rather unsatisfactory results were shown for this method [7, 8]. According to the literature the average rate of repeated revision of the acetabular component ranges between 20 and 36% at 10 years after the initial revision [9–11]. It should be noted that there is no clear consensus regarding the best method/option for performing revision arthroplasty in patients with IIIB defects. There is an increasing number of scientific publications concerning the use of custom-designed 3D components, which make it possible not only to achieve stable fixation and connect the pelvic bones but also to restore hip joint biomechanics [12–16].

Advances in radiology and computed tomography made it possible to visualize the entire three-dimensional anatomy, expanding our horizons [17]. However, the rate of complications after implantation of custom-designed structures remains high and, of course, today an important and difficult task is to assess proper implantation of the construction according to preoperative planning [18].

The study aimed to evaluate the positioning of 3D acetabular implants after revision hip arthroplasty and its impact on clinical and functional outcomes.

## Materials and methods

Inclusion criteria:

- Loosening of the acetabular component of the hip joint implant with bone defect type IIIB according to W.G. Paprosky’s classification of acetabular bone defects [19];
- Performing the second stage of treatment of periprosthetic infection (removal of the spacer, implantation of endoprosthetic components).

Non-inclusion criteria:

- HIV, drug addiction;
- Deep periprosthetic hip joint infection;
- Severe somatic pathology requiring active treatment, being the contraindication to surgical intervention or significantly increasing the surgical risk;
- Lack of CT scan of the pelvic bones in the postoperative period in patients who underwent revision arthroplasty with custom-designed 3D acetabular implants.

Exclusion criteria:

- Fistulous type of periprosthetic infection;
- Decompensated somatic pathology before surgical treatment;
- Lack of consent to participate in the study.

According to the above criteria, we analyzed treatment results in 48 patients with bone defect types IIIA and IIIB, including pelvic bone dissociation, who underwent revision hip arthroplasty in the setting of the City Clinical Hospital named. S.P. Botkin. A prospective study was conducted from 2017 to 2023. Revision endoprosthesis replacement due to aseptic loosening of endoprosthetic components was performed in 30 (62.5%) cases and as a second stage of periprosthetic infection treatment in 18 cases (37.5%). Before performing revision surgery, all patients underwent a comprehensive periprosthetic infection diagnostic algorithm, which included mandatory puncture followed by microbiological study [Parvizi J., 2018] [20]. There were 7 (14.6%) patients with type IIIA acetabular bone defects, and 41 (85.4%) patients with type IIIB defects, of which 22 (53.6%) had pelvic bones dissociation.

## Techniques for preoperative planning and postoperative assessment of custom-designed implant positioning

In the first stage, we assessed pelvic bones with plain radiographs, and patients with significant bone defects of acetabular region routinely underwent ≤ 0.6-mm-thick CT (computed tomography) scanning with image recording in DICOM format; with subsequent decision-making to perform surgical intervention using custom-designed components. The planning of surgical intervention and the production of custom-designed implants was carried out jointly with designers from the “Additive Technologies Laboratory”.

**Figure 1:**
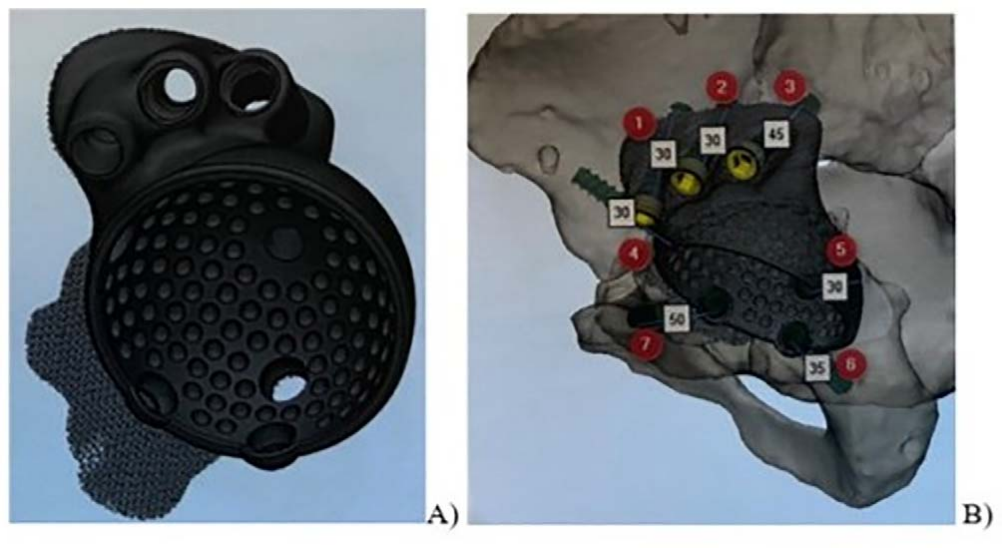
Planning for revision hip arthroplasty. (A) virtual model of the custom-designed acetabular component. (B) Scheme of 3D component fixation in the acetabulum.

In the second stage, the design engineer using the specialized software Materialize Mimics Medical segmented the obtained CT data, yielding a virtual 3D model of the pelvic defect. Based on the pelvic virtual model, together with the operating surgeons, virtual planning of acetabular component rotation center positioning was performed, the diameter and position angles of the component hemisphere were selected, and the implant fixation schemes were approved. Based on the collaboratively agreed data, the design engineer generated the component design using “Materialize 3-matic Medical” software and developed supporting operational documentation for final approval (Figures 1A and 1B).

**Figure 2:**
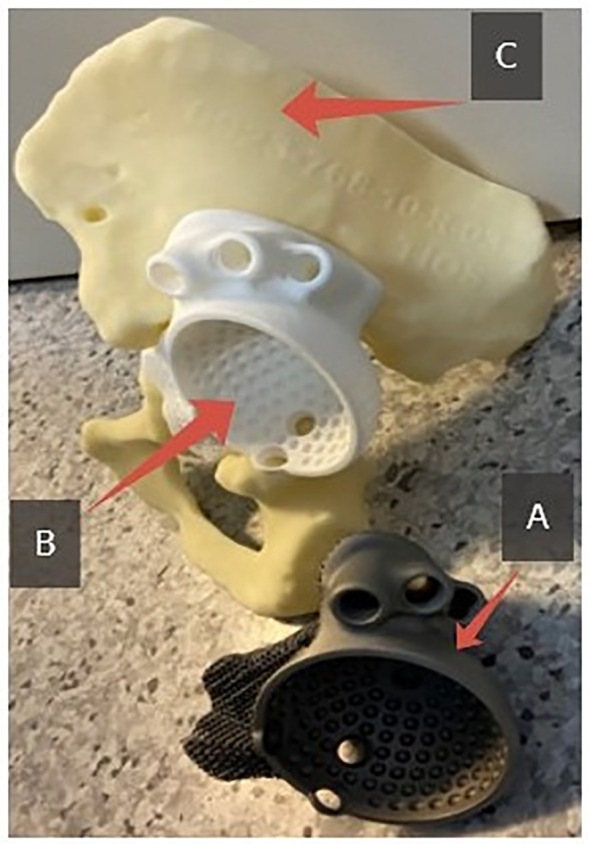
Kit for revision acetabular arthroplasty: (A) custom-designed acetabular component made of titanium alloy. (B) Component fitting template. (C) Anatomical model of the pelvic bone.

After approval, the anatomical model was printed including the areas of the pelvic bone defect, a fitting template, and a titanium alloy custom-designed acetabular component (Figure 2).

Next, standard surgical treatment was carried out, which consisted of removing the loose component or spacer, followed by implantation of a custom-designed implant, implantation of an endoprosthesis, and closing the surgical wound.

Individual 3D components of the acetabulum components have already been designed at the planning stage with an anteversion of 20 degrees and an inclination of 45 degrees. Each component personifies the contour of the bone defect and is fixed cementless with bone screws. Then, in the 3D acetabulum component itself, the cement fixation cup of the double mobility system was fixed, which made it possible to eliminate the possibility of deviation of the acetabular part of the initially set parameters. Therefore, in this study, the task was precisely the area of contact, which could be critical for the appearance of complications and deterioration of the clinical and functional results.

## Method for assessing implant positioning after implantation

In the early postoperative period, all patients underwent a CT scan with a slice thickness of ≤0.6 mm, recorded in DICOM format, and these results were referred further to the design engineers of the “Additive technology laboratory” in order to segment this CT scan results and to obtain virtual postoperative 3D models of the pelvic bones and the installed custom-designed acetabular component. 3D models in the designing and production of custom-designed models using additive technologies were made in STL format. STL (stereolithography) is a file format widely used for storing three-dimensional models of objects for use in additive technologies.

**Figure 3:**
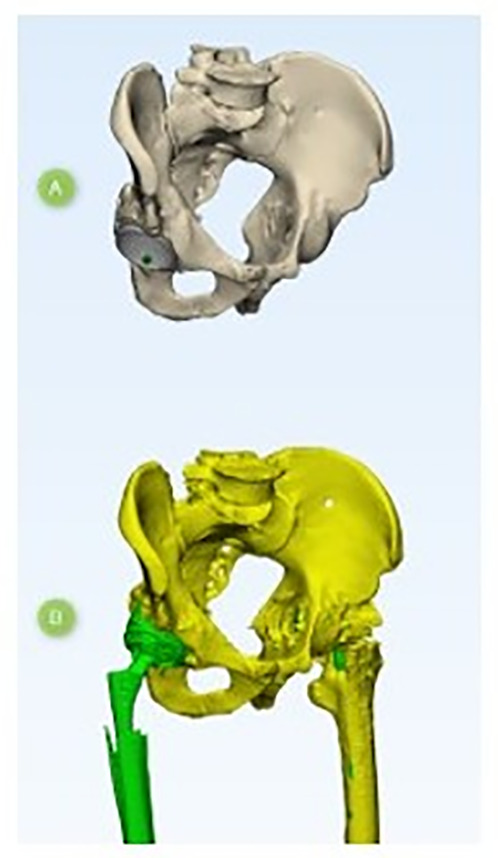
Spatial position of the models: (A) Preoperative 3D models. (B) Post-operative 3D models.

**Figure 4:**
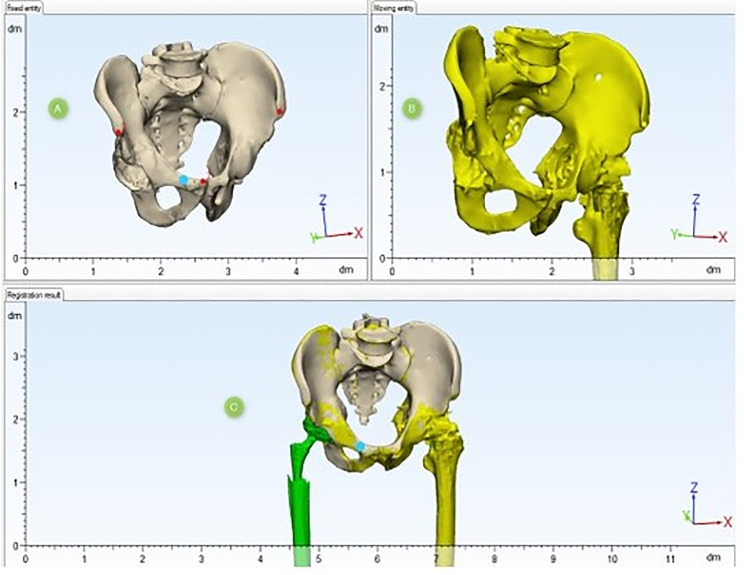
Models alignment using pelvic bones reference points: (A) Preoperative fixed model of the pelvis. (B) Postoperative moving model of the pelvis. (C) Spatial alignment of 3D models.

**Figure 5:**
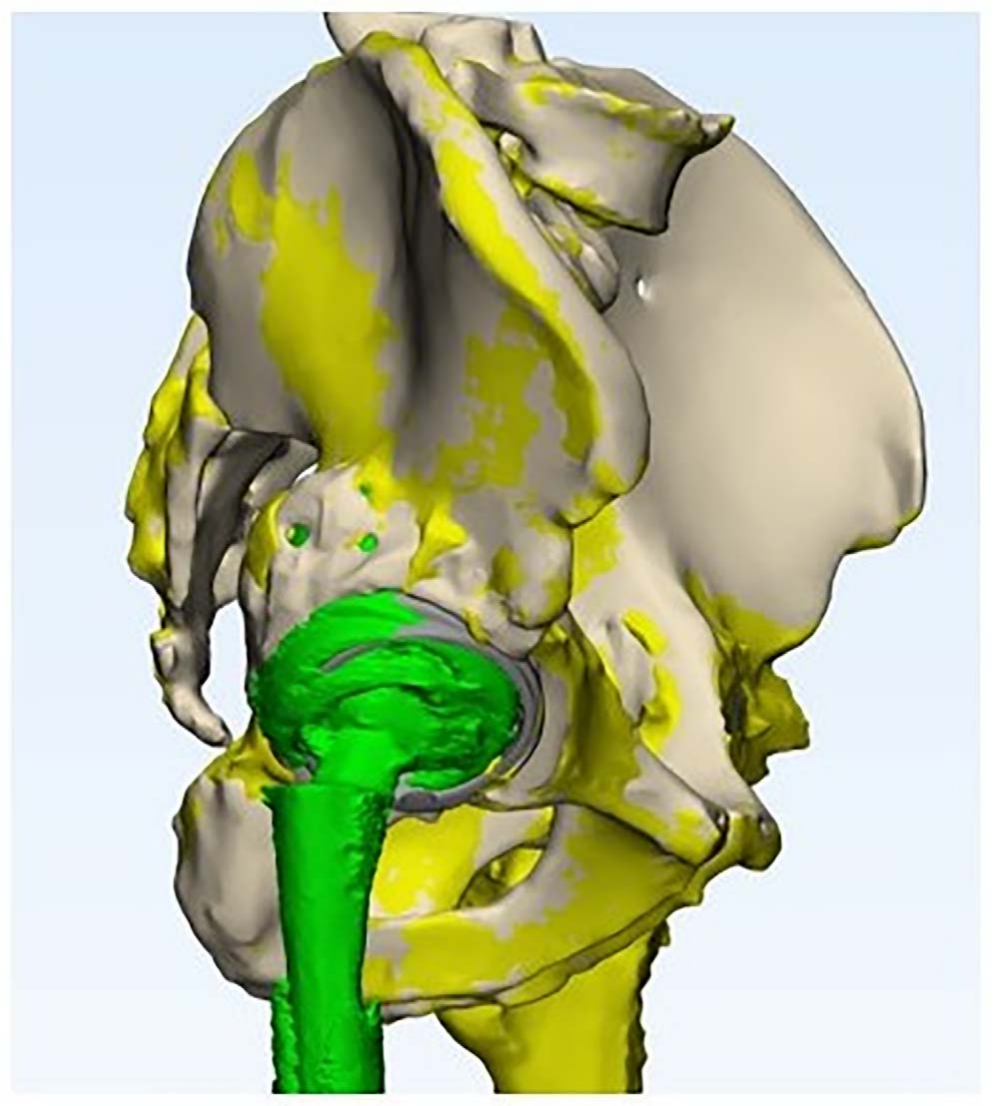
Spatial alignment of 3D models.

The technique for comparing preoperative and postoperative models consists of spatial alignment at several reference points of the pelvic bones (Figure 3). Spatial alignment of objects was based on the anthropometric features of pelvic bone structure. For accurate alignment, the minimum requirement is to combine at least three spatial points. First, a fixed reference point was selected on a model; in our case, it was a preoperative model; then, this point was marked on the postoperative model, which moved in space together with the model of the acetabular component, and thus at least three iterations were performed. Figure 4 shows the method for selecting reference points of the pelvic bones; the points are selected in such a way that in both models the selection of points is accurate. When performing CT segmentation in the preoperative and postoperative period, possible errors are to be taken into account, which may be due to the presence of artifacts from the glow of metal implants on post-revision CT scans (Figure 5).

Next, based on the comparison results, the percentage of adherence of porous structures of the acetabular component versus the planned one was calculated. For this purpose, using the software, the surface area adhering to the bone structures in the preoperative models, as well as the surface area adhering to the bone structures in the postoperative models were calculated. An example of calculating the percentage of adherence is given in Table 1.

**Table 1:** Example of calculating percentage of adherence.

Planned	
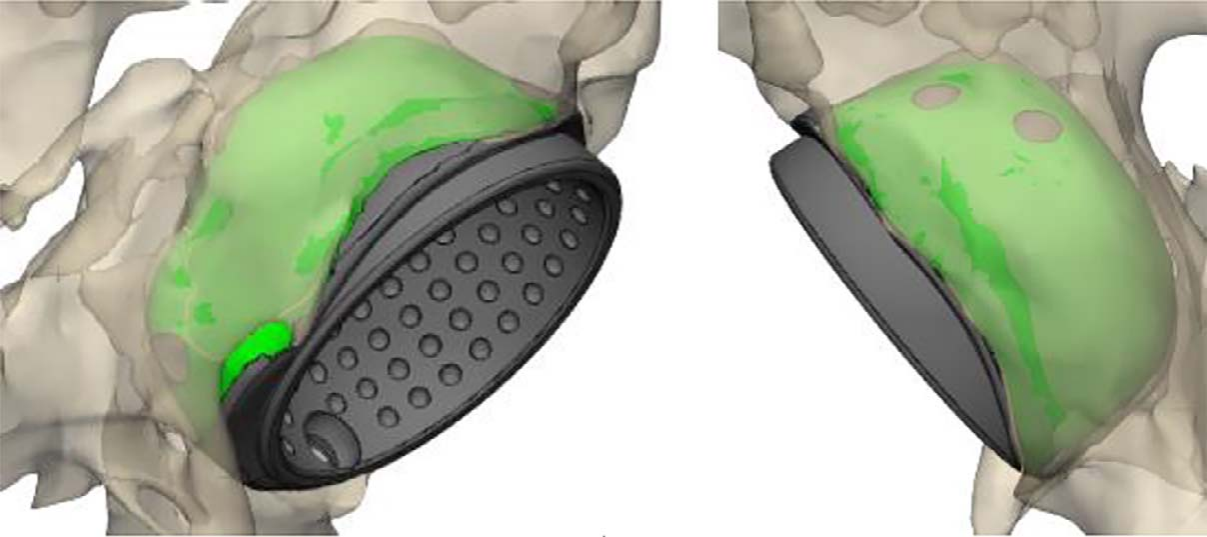	
Surface area, mm^2^	6838
Actual	
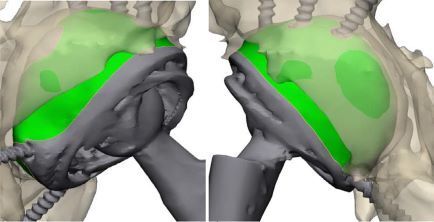	
Surface area, mm^2^	6505
Result (%)	95,13016

## Statistical analysis

Quantitative data were assessed for compliance with normal distribution using the Shapiro-Wilk test.

Quantitative data with a normal distribution were described using arithmetic means (M) and standard deviations (SD), 95% confidence interval limits (95% CI).

In the absence of normal distribution, quantitative data were described using the median (Me) and lower and upper quartiles (Q1–Q3).

Categorical data were described using absolute values and percentages. 95% Confidence intervals for percentages were calculated using the Clopper–Pearson method.

Comparison of two groups according to quantitative parameters with a normal distribution provided that the variances were equal, was performed using the Student’s *t*-test.

The direction and strength of the correlation between two quantitative indicators were assessed using Spearman’s rank correlation coefficient (in case of no normal parameter distribution).

A prognostic model characterizing the dependence of a quantitative variable on factors was developed using the linear regression method. To assess the diagnostic significance of quantitative characteristics in predicting a specific outcome, the ROC curve analysis method was used. The separating value of a quantitative characteristic at the cut-off point was determined using the Youden index’s highest value. Differences were considered statistically significant at *p* < 0.05.

## Evaluation of results

For statistical analysis, the following parameters were used: type of bone defect of the acetabulum according to W.G. Paprosky classification, the presence of pelvic bones dissociation, the use of additional flanges for fixing 3D components, the percentage of adherence of porous structures of 3D acetabular component versus the planned one. We assessed pain and also clinical and functional results before surgical treatment and after 3, 6, and 12 months, and then once a year using the WOMAC, Harris Hip Score, and VAS scales. The rates and types of postoperative complications were analyzed: aseptic loosening, endoprosthesis instability, and the development of PJI.

## Results and discussion

### Results

**Figure 6:**
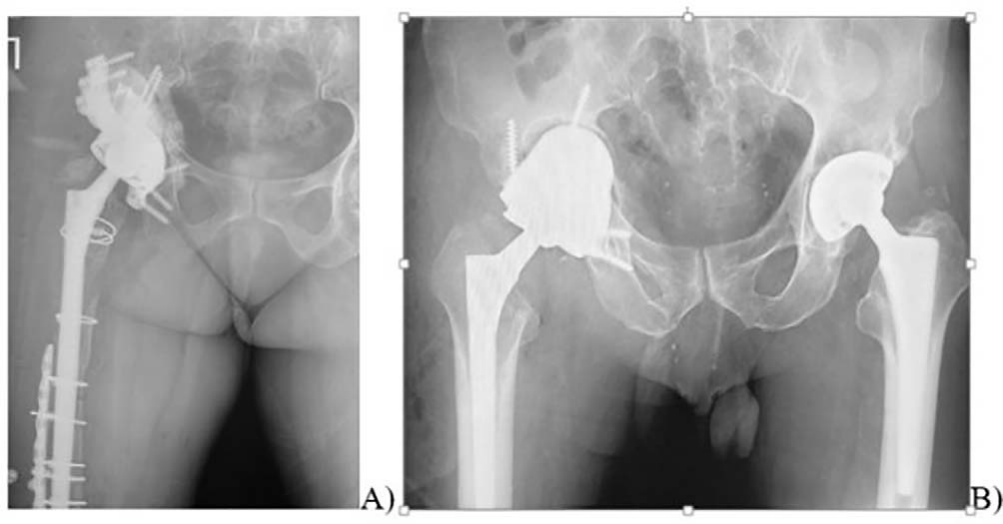
Radiographs of patients after revision hip arthroplasty: (A) 3D acetabular component with additional three-flange fixation. (B) 3D acetabular component without flanges (right).

The mean patient age was 62.2 years (ranging from 46.4 to 78.3 years). The mean follow-up duration was 42 months (ranging from 6 to 82 months). To reconstruct acetabular bone defect, in 28 (58.3%) cases we used 3D components with additional three-flange fixation, and in 20 (41.7%) cases we used 3D components without additional flanges (Figure 6), (Table 2).

**Table 2:** Descriptive statistics of categorical variables.

Parameter	Category	Abs.	%	95% CI
Defect type	IIIA	7	14.6	4.0–32.7
	IIIB	41	85.4	67.3–96.0
Pelvic bones dissociation	Yes	22	45.8	30.6–69.4
	No	26	54.2	30.6–69.4
Flange	Yes	28	58,4	30.6–69.4
	No	20	41,6	30.6–69.4

Table 3 shows the percentage of adherence to porous structure versus the planned adherence, and shows clinical and functional results.

**Table 3:** Descriptive statistics of quantitative variables.

Parameter	*M* ± SD/Me	95% CI/Q_1_–Q_3_	Min	Max
Matching portion, Me	63.99	50.00–93.27	25.44	99.66
HHS, *M* ± SD (score)	80.54 ± 4.73	78.70–82.37	68.00	87.00
WOMAC, *M* ± SD (score)	9.46 ± 5.73	7.24–11.69	0.00	23.00
VAS, Me (score)	0.00	0.00–1.00	0.00	3.00

We analyzed the matching portion by the type of acetabular bone defect according to W.G. Paprosky’s classification (Table 4).

**Table 4:** Analysis of matching portion by the type of defect.

Parameter	Categories	Matching portion			*p*
		*M* ± SD	95% CI	*n*	
Defect type	IIIA	61.57 ± 26.08	20.07–103.07	7	0.700
	IIIB	66.77 ± 24.47	56.44–77.10	41	

When assessing the matching portion by the defect type, we found no statistically significant differences (*p* = 0.700) (Student’s *t*-test) (Table 5).

**Table 5:** Threshold values for matching portion.

Threshold	Sensitivity (Se), %	Specificity (Sp), %	PPV	NPV
68.05	50.0	75.0	92.3	20.0
64.44	50.0	50.0	85.7	14.3
63.53	58.3	50.0	87.5	16.7
57.00	70.8	50.0	89.5	22.2

The area under the ROC curve was 0.583 ± 0.150 (95% CI: 0.290–0.877). The resulting model was not statistically significant (*p* = 0.599).

The threshold value for the matching portion at the cut-off point, which corresponded to the highest value of the Youden index, was 97.033. IIIB was predicted when the matching portion was higher than or equal to this value. Model sensitivity and specificity were 25.0% and 100.0%, respectively.

We analyzed the matching portion depending on pelvic bone dissociation (Table 6).

**Table 6:** Analysis of matching portion depending on pelvic bone dissociation.

Parameter	Categories	Matching portion			*p*
		M ± SD	95% CI	n	
Pelvic bones dissociation	Yes	68.73 ± 21.98	56.04–81.42	22	0.565
	No	63.32 ± 26.92	47.78–78.87	26	

When comparing the matching portion depending on pelvic bone dissociation, we found no significant differences (*p* = 0.565) (Student’s *t*-test).

The area under the ROC curve was 0.548 ± 0.110 (95% CI: 0.332–0.765). The resulting model was not statistically significant (*p* = 0.662).

The threshold value for the matching portion at the cut-off point, which corresponded to the highest value of the Youden index, was 44.998. “No” was predicted when the matching portion value was below this value. Model sensitivity and specificity were 28.6% and 92.9%, respectively (Table 7).

**Table 7:** Threshold values for matching portion.

Threshold	Sensitivity (Se), %	Specificity (Sp), %	PPV	NPV
68.05	57.1	50.0	53.3	53.8
64.44	50.0	50.0	50.0	50.0

We analyzed the matching portion depending on the flange presence (Table 8).

**Table 8:** Analysis of matching portion depending on the flange presence.

Parameter	Category	Matching portion			*p*
		M ± SD	95% CI	*n*	
Flange presence	Yes	68.51 ± 23.93	54.69–82.32	28	0.598
	No	63.55 ± 25.26	48.96–78.13	20	

When analyzing the matching portion depending on the flange presence, we found no statistically significant differences (*p* = 0.598) (Student’s *t*-test).

The area under the ROC curve was 0.589 ± 0.109 (95% CI: 0.376–0.803). The resulting model was not statistically significant (*p* = 0.421).

The threshold value for the matching portion at the cut-off point, which corresponded to the highest value of the Youden index, was 57.624. Status “No” was predicted when the matching portion was below this value. Model sensitivity and specificity were 50.0% and 78.6%, respectively (Table 9).

**Table 9:** Threshold values for matching portion.

Threshold	Sensitivity (Se), %	Specificity (Sp), %	PPV	NPV
68.30	64.3	50.0	56.2	58.3
68.05	57.1	50.0	53.3	53.8
64.44	57.1	57.1	57.1	57.1
63.53	50.0	64.3	58.3	56.2
57.62	50.0	78.6	70.0	61.1

We conducted a correlation analysis of the relationship between the Percentage of Match and WOMAC (Table 10).

**Table 10:** Results of correlation analysis of the relationship percentage of agreement and WOMAC.

Parameter	Correlation relationship characteristics		
	*ρ*	Strength of relationship according to Chaddock scale	p
Matching portion – WOMAC	−0.186	Weak	0.344

When assessing the relationship between WOMAC and the matching portion, a weak inverse relationship was found.

The observed dependence of WOMAC on the matching portion is described by the paired linear regression equation:

$$Y_{\mathrm{WOMAC}}=-0.033\times X_{\mathrm{matching}\;\mathrm{portion}}+11.639.$$

When the matching portion is increased by 1, a decrease in WOMAC by 0.033 points is expected. The resulting model explains 1.9% of the observed WOMAC variance.

We also performed a correlation analysis of the matching portion and HHS relationship (Table 11).

**Table 11:** Results of matching portion and HHS relationship correlation analysis.

Parameter	Correlation relationship characteristics		
	*ρ*	Strength of relationship according to Chaddock scale	*p*
Matching portion – HHS	0.055	No relationship	0.783

No association was found between HHS and the matching portion.

The observed dependence of HHS on the matching portion is described by the pairwise linear regression equation:

$$Y_{\mathrm{HHS}}=0.017\times X_{\mathrm{matching}\;\mathrm{portion}}+79.42.$$

When the matching portion is increased by 1, a 0.017 point increase of HHS is expected. The resulting model explains an observed 0.8% HHS variance.

We also conducted a correlation analysis for the relationship between the matching portion and VAS (Table 12).

**Table 12:** Results of matching portion and VAS relationship correlation analysis.

Parameter	Correlation relationship characteristics		
	*ρ*	Strength of relationship according to Chaddock scale	*p*
Matching portion – VAS	−0.134	Weak	0.497

We found a weak inverse relationship between VAS and the matching portion.

The observed relationship between VAS and the matching portion is described by the paired linear regression equation:

$$Y_{\mathrm{VAS}}=-0.003\times X_{\mathrm{matching}\;\mathrm{portion}}+0.928.$$

When the matching portion is increased by 1, a 0.003-point decrease in VAS is expected.

The resulting model explains an observed 0.6% VAS variance.

We did not achieve a statistically significant difference when using additional flanges and clinical and functional results. However, in two cases we faced aseptic loosening in patients using flanges (migration of fixing screws). It is worth emphasizing that in no case were we able to install an implant with 100% adherence to porous structure compared to preoperatively planned adherence. We noted that according to the WOMAC and VAS scales, increasing the contact area of the components there was a slight statistical difference in the improvement of clinical and functional results and the reduction of pain.

All complications that we registered were in cases with components adhering to the bone no more than 68%. We registered a total of 8 (16.6%) complications, including:

- Aseptic loosening of component (2 cases, 4.2%), which were diagnosed 6 months after surgery; at the time of writing the manuscript of this article, new custom-designed components were made for these patients, and revision arthroplasty was scheduled (Figure 7);
  **Figure 7:**
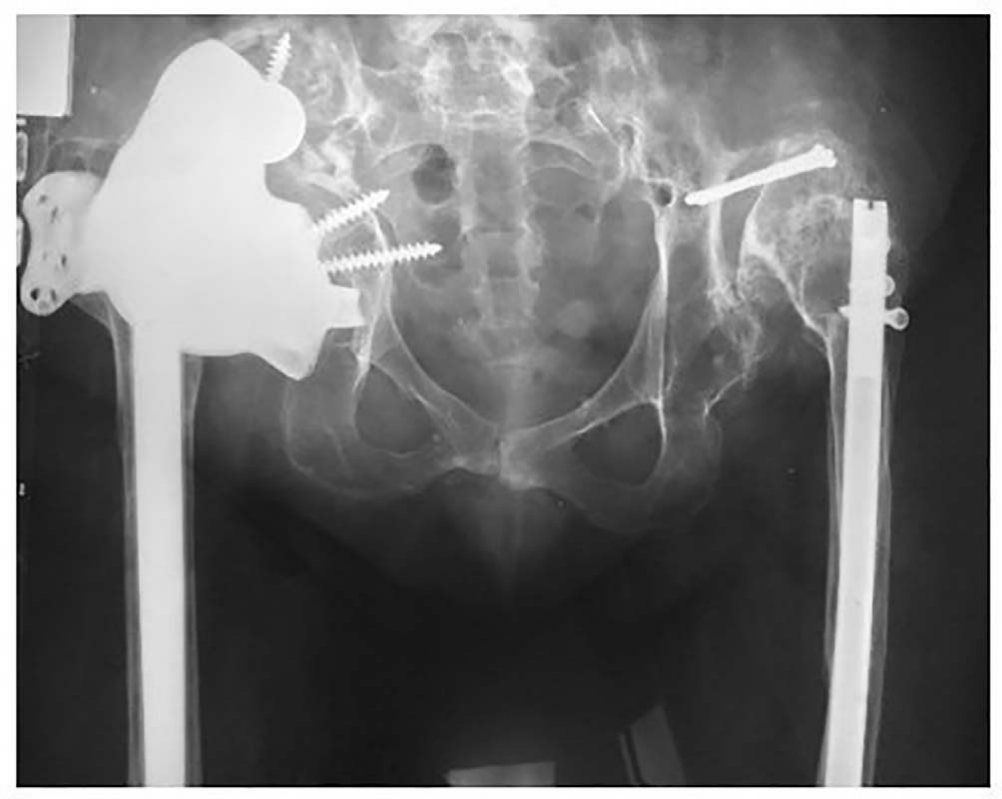
Loosening of right hip joint 3D structure, migration of the fixing screws.
- Joint instability (3 cases, 6.2%); in one case it was treated conservatively, via closed reduction of dislocation; and in two cases it was treated surgically, via open reduction with increasing the head length (Figure 8);
  **Figure 8:**
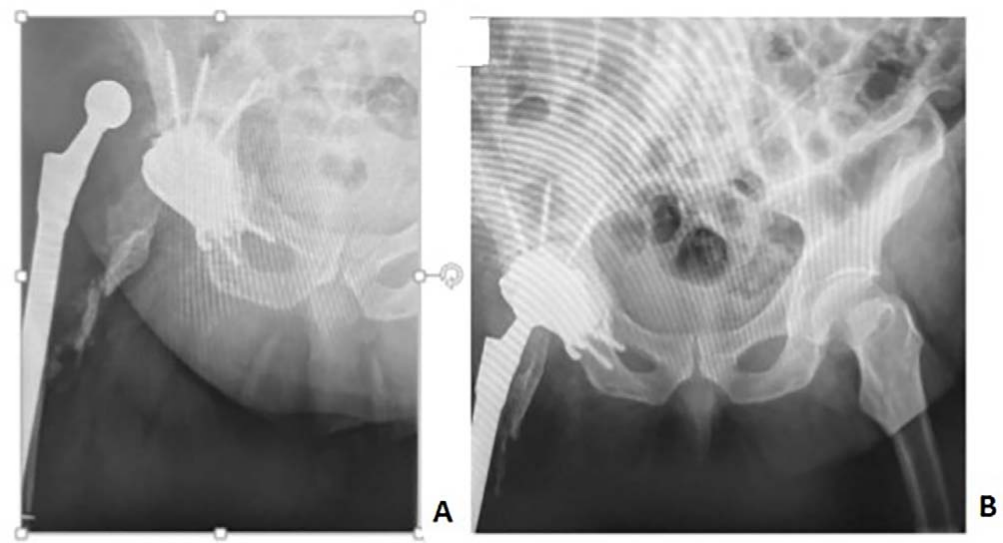
3D implant dislocation. (A) X-rays of the right hip joint with implant dislocation. (B) X-rays of pelvic bones, after open reduction of dislocation with increasing endoprosthesis head length.
- Early periprosthetic infection (3 cases, 6.2%); in all three cases, debridement was performed; and at the time of writing the manuscript of this article there were no signs of relapse and loosening of components in these patients.

### Discussion

Even for the most experienced surgeons, revision hip arthroplasty is quite a difficult task, especially in cases of progressive acetabular bone loss. It is especially difficult to perform acetabular reconstruction in patients with types IIIA and IIIB defects according to W.G. Paprosky classification [21]. In most cases, these patients void of any landmarks for adequate component fixation, which affects the accuracy of implant installation and the corresponding restoration of the rotation center [12, 15].

There is an increasing number of literature reports on the successful use of custom-designed implants for patients with massive bone defects in the acetabular area [17, 22–25]. It is worth noting that in a 2023 review article, D. Broekhuis et al. reported a 24% total rate of complications after acetabular revision surgery using custom-designed 3D acetabular components in patients with W.G. Paprosky IIIA and IIIB defects, being 12% four years after implantation [26]. In our study, the rate of complications was 16.6%, which is comparable with the literature data.

Careful preoperative preparation and planning for each revision are mandatory for the successful completion of revision arthroplasty [27]. Certainly, accurate preoperative planning can prevent complications such as periprosthetic fractures or early loosening of implanted endoprosthetic components [28]. Because a 3D implant has a personalized geometry designed for each defect, it is impossible to assess the accuracy of its positioning on postoperative X-rays, therefore it is necessary to perform CT with subsequent 3D modeling [29, 30].

Currently, 3D modeling software and tools for segmentation and reduction of metal artifacts allow the use of computed tomography images to assess bone defects in 3D format and to confirm the accuracy and reliability of the use of innovative technologies [29, 31].

Zampelis and Flivik, in their study, reported a high opinion of the positioning of individual acetabular components one year after surgical treatment. However, a limitation of this study is that the authors evaluated only 10 patients and only those with W.G. Paprosky type IIIA acetabular bone defects [15].

PubMed, Web of Science, and EMBASE contain reports devoted to the assessment of 3D components positioning in the postoperative period. However, in most cases, authors evaluate only the angles of anteversion and inclination of components. However, there are few reports assessing specifically the adherence of porous structures of custom-designed acetabular components.

For example, Romagnoli et al., reported three cases where after revision arthroplasty, using 3D acetabular components, patients underwent CT to assess the adherence of the custom-designed components to the bone compared to preoperatively planned positioning. This article demonstrates three cases with W.G. Paprosky type IIIA (2 cases) and IIIB (1 case) defects, and the maximum observation period of up to 4 years, which also indicates the limitations of this study. The authors reported that according to postoperative CT results they also failed to achieve more than 74.1% adherence of the porous part of the individual acetabular component to the bone [32]. Our study demonstrated more promising data: in 50% of cases, it was possible to achieve more than 67% adherence to a porous portion of 3D components.

Some authors describe that a smaller contact area between the implant and the bone may be due to the presence of scar tissue on the bone surface, the lack of special instruments for better visualization of the entire defect in the wound [33], as well as the very removal of a stable component, even using special instrumentation can lead to additional bone loss [29, 34]. Another significant factor is the time lag from the moment of performing a CT scan, planning surgical treatment, manufacturing the implant, and carrying out the surgical intervention itself because the bone tissue defect itself can only increase over time [32].

Our study demonstrated the stability of custom-designed 3D acetabular components, even at a low percentage of bone contact compared to the preoperatively planned contact area. However, long-term follow-up of this patient pool is necessary to assess implant stability.

## Conclusion

When the custom-designed acetabular 3D components adhered to the bone by more than 68%, we did not register a single complication in the postoperative period, and when custom-designed acetabular 3D components adhered to the bone by less than 68%, a total of 8 (16.6%) complications were registered. However, in two cases we do not suspect the presence of infectious complications to be associated with component adherence to the bone.

The presence of flanges on the implant does not increase the area of its contact with the bone from what was planned at the preoperative stage. We also registered two cases of aseptic loosening of 3D acetabular components with flanges. At this time point of follow-up, we did not notice a statistical difference in clinical and functional results depending on the positioning of the implant according to the Harris hip score. However, according to the WOMAC and VAS scales, there was a minimal tendency to improve the clinical and functional outcome and to reduce pain, however, this result cannot be interpreted as having clinical significance and most likely it is due to a small sample size.

## Limitation of this study

This study was limited by an insufficiently long follow-up period – mead duration of 42 months – and a small sample size due to the small number of clinical cases. We also did not evaluate the angles of anteversion and inclination in the postoperative period compared to the preoperatively planned but only assessed the area of contact of the porous structure of the 3D component with the bone.

## Data Availability

The data that support the findings of this study are available from the corresponding author, upon reasonable request.
